# Ovarian borderline tumors in the 2014 WHO classification: evolving concepts and diagnostic criteria

**DOI:** 10.1007/s00428-016-2040-8

**Published:** 2016-12-27

**Authors:** Steffen Hauptmann, Katrin Friedrich, Raymond Redline, Stefanie Avril

**Affiliations:** 1 Department of Pathology, GROW School for Oncology and Developmental Biology, Maastricht University Medical Center, Maastricht, The Netherlands; 2 Institute of Pathology, University Hospital Dresden, Dresden, Germany; 3 Department of Pathology, Case Western Reserve University School of Medicine, University Hospitals Cleveland Medical Center, Cleveland, OH USA

**Keywords:** Borderline tumor, Ovary, Diagnostic criteria, WHO classification 2014

## Abstract

Borderline ovarian tumors (BOT) are uncommon but not rare epithelial ovarian neoplasms, intermediate between benign and malignant categories. Since BOT were first identified >40 years ago, they have inspired controversies disproportionate to their incidence. This review discusses diagnostic criteria for the histologic subtypes of BOT, highlighting areas of diagnostic challenges, ongoing controversies, and changes in terminology implemented by the recent 2014 WHO Classification of Tumours of the Female Genital Organs. Emerging knowledge supports the notion that subtypes of borderline ovarian tumors comprise distinct biologic, pathogenetic, and molecular entities, precluding a single unifying concept for BOT. Serous borderline tumors (SBT) share molecular and genetic alterations with low-grade serous carcinomas and can present at higher stages with peritoneal implants and/or lymph node involvement, which validates their borderline malignant potential. All other (non-serous) subtypes of BOT commonly present at stage I confined to the ovary(ies) and are associated with overall survival approaching that of the general population. An important change in the WHO 2014 classification is the new terminology of non-invasive implants associated with SBT, as any invasive foci (previously called “invasive implants”) are now in line with their biological behavior considered peritoneal low-grade serous carcinoma (LGSC). The controversy regarding the terminology of non-serous borderline tumors, called by some pathologists “atypical proliferative tumor” in view of their largely benign behavior, has not been resolved. The concepts of intraepithelial carcinoma and microinvasion may evolve in further studies, as their presence appears to have no prognostic impact and is subject to considerable inter-observer variability.

## Introduction

Borderline ovarian tumors (BOT) are neoplasms of epithelial origin characterized by up-regulated cellular proliferation and the presence of slight nuclear atypia but without destructive stromal invasion [1]. This group of tumors was first described by Taylor in 1929 as “semi-malignant” ovarian tumors with peritoneal involvement but surprisingly good prognosis and subsequently recognized by the International Federation of Gynecology and Obstetrics (FIGO) in 1971 as tumors of “low malignant potential” distinct from ovarian carcinomas [2] followed by the WHO in 1973 [3]. The current 2014 WHO Classification of Tumours of the Female Genital Organs uses the term “borderline tumor” interchangeable with “atypical proliferative tumor”—a terminology that was discouraged in the previous WHO classification [4], whereas the previously advocated synonym “tumor of low malignant potential” is no longer recommended [5].

Six histologic subtypes of BOT are distinguished based on the epithelial cell type, similar to invasive carcinomas, comprising serous (50%) and mucinous (45%), and less common subtypes including endometrioid, clear cell, seromucinous, and borderline Brenner tumor [1, 6]. Although the distinction of serous or mucinous BOT from frankly malignant tumors with destructive stromal invasion does not usually pose a diagnostic problem, BOT can be associated with microinvasion, intraepithelial carcinoma, lymph node involvement, and non-invasive peritoneal implants [7] and establishing the correct diagnosis can be challenging in these cases. The diagnostic criteria are less well defined for the uncommon histologic subtypes and sometimes hampered by subjectivity. The distinction of BOT from its benign adenomatous counterparts is equally important, and overdiagnosis should be avoided, since it has important clinical implications regarding staging and follow-up. A workshop sponsored by the National Institutes of Health Office of Rare Diseases in 2003 provided consensus for many of the currently accepted criteria defining various aspects of BOT [1].

The vast majority of BOT are limited to the ovary(ies) at presentation with 75% being diagnosed at FIGO stage I, compared to only 10% of ovarian carcinomas diagnosed at an early stage. They generally have an excellent prognosis with a 10-year survival of 97% for all stages combined [8–10], although recurrences and malignant transformation can occur. Standard treatment includes complete surgical resection and surgical staging including omentectomy, peritoneal biopsies, cytology of peritoneal washings, and appendectomy in case of mucinous BOT [10, 11]. Adjuvant chemotherapy is not indicated [12, 13].

This review summarizes pertinent diagnostic criteria for all six different histologic subtypes of BOT, with particular emphasis on areas of ongoing controversy and changes implemented by the recent WHO 2014 classification compared to previous classifications.

### Serous borderline tumor

Approximately 50–55% of BOT belong to this subtype (synonymous “atypical proliferative serous tumor”) [5]. Molecular analyses have demonstrated that serous borderline tumors (SBTs) harbor similar molecular and genetic alterations as low-grade serous carcinomas (LGSC) [14–17]. In some cases, a continuous tumor progression from cystadenomas and BOT to low-grade carcinomas may exist, and co-existing areas of SBT were observed in 30 out of 50 LGSC in a series by Malpica et al. [18]. KRAS and BRAF mutations are each present in about 30% of SBT, usually in a mutually exclusive fashion [14, 19–21]. In contrast, p53 mutations are almost exclusively found in high-grade serous ovarian carcinomas [15, 22]. While the fallopian tube has been established as the site of origin for some high-grade serous carcinomas, SBTs were historically presumed to originate in the ovarian cortex or peritoneal surface. Recent reports showing a higher frequency of PAX2-negative secretory cell outgrowths (SCOUT) in the fallopian tubes of women with SBT may warrant further study [23].

**Fig. 1:**
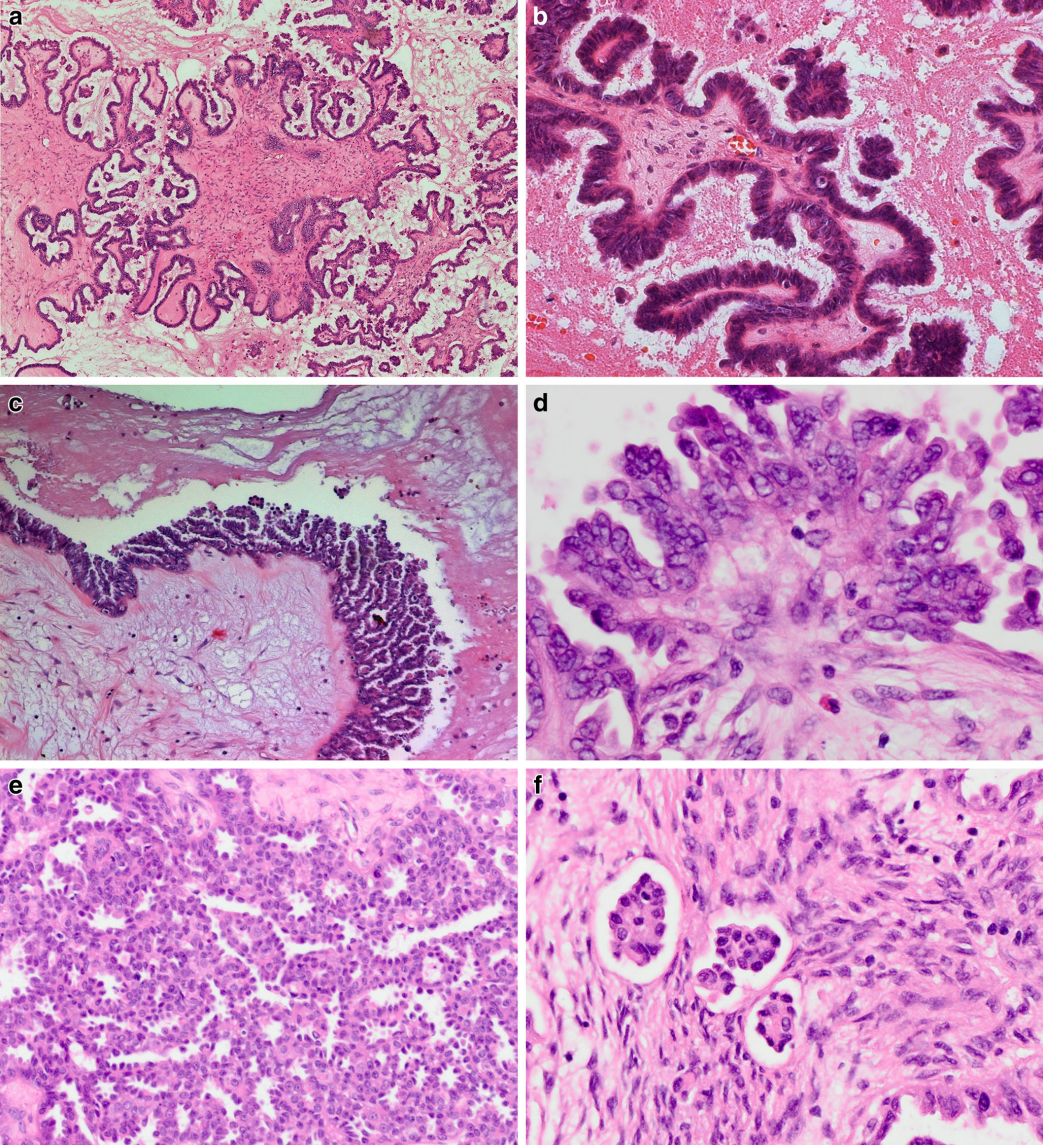
SBT with hierarchically branched papillae (**a**) covered by a single-layered or multilayered epithelium with pseudopapillary proliferations and secretory or ciliated serous differentiation (**b**). Micropapillary SBT demonstrates non-hierarchical “Medusa-like” branching and a more cellular stroma (**c**). Its epithelium is more cuboidal with “nuclear atypia greater than that allowed for conventional SBT” and often containing small nucleoli (**d**). Some micropapillary SBTs have a predominant cribriform epithelial proliferation (**e**). Microinvasion is characterized by small epithelial cell groups surrounded by retraction artifacts within a cell-rich fibroblastic stroma (**f**)

On gross pathologic examination, SBTs are unilocular or multilocular cystic tumors with or without epithelial proliferations on the outer tumor surface. About one third of SBTs are bilateral. The histology of BOT is characterized by hierarchically branching papillae and pseudopapillae with paucicellular, edematous, or hyalinized fibrous stroma, lined by architecturally complex epithelial proliferations (Fig. 1a). The epithelial cells are typically columnar, resembling secretory cells of the fallopian tube, admixed with variable numbers of ciliated cells. There is mild to moderate nuclear atypia, hyperchromasia, epithelial multilayering, and cell detachment (“tufting”) into the lumen [6, 24] (Fig. 1b). Most authors, including the WHO 2014 classification, agree that >10% borderline histology within a cystadenoma or cystadenofibroma qualifies as BOT. In contrast, serous cystadenomas with foci qualifying as SBT in <10% of the epithelial volume are designated “cystadenoma/fibroma with focal epithelial proliferation” [1, 25] [5]. By immunohistochemistry, SBTs are characterized by expression of WT1, PAX8, Bcl-2, estrogen and progesterone receptor [26–28]

.

A recent retrospective study by the authors demonstrated that overdiagnosis of cystadenoma/fibroma as SBT has been a relevant clinical problem in the past, albeit in a low percentage of cases. Out of 81 consecutive cases diagnosed as BOT over a 10-year period at a single tertiary center (1998–2008), the diagnosis of SBT was rejected due to a diagnosis of serous cystadenoma/fibroma in 7 (9%) patients [29]. This cohort of 81 patients was part of a larger multicenter study, which confirmed that an overdiagnosis of borderline tumor had been made in 11.5% (92/803) of patients [30].

#### Microinvasion

The term “microinvasion” has been refined in the recent WHO 2014 classification and is now limited to isolated rounded eosinophilic cells or cell clusters within the stroma, with a cytomorphology resembling the epithelial cells lining the surface of the papillae. Microinvasive foci are often surrounded by retraction spaces and a stroma rich in fibroblasts and cannot exceed 5 mm in the largest linear dimension [5, 25, 31, 32] (Fig. 1f). In contrast, solid nests or cribriform glands cytologically resembling low-grade serous carcinoma, previously also classified as microinvasion when measuring <5 mm, are now designated LGSC regardless of their size. These small invasive carcinomas may be diagnosed as “microinvasive carcinoma” [5] and are characteristically surrounded by desmoplastic stroma with abundant collagenous extracellular matrix without significant inflammation or fibroblast proliferation.

The nature of epithelial cells in microinvasive foci has only recently been started to be elucidated. Kurman’s group demonstrated a lower intensity of WT1, as well as estrogen and progesterone receptor expression, and a lower Ki67 index in microinvasive foci compared to the columnar/cuboidal tumor cells covering the papillary surfaces in 37 patients with SBT. Together with morphologic evidence of apoptosis, these findings suggest terminal differentiation and/or senescence in microinvasive cells [33]. Nevertheless, this does not fully explain why these cells become entrapped within stroma. In addition, a higher rate of microinvasion has been observed in SBT diagnosed during pregnancy [32, 34], suggesting possible influence by hormonal factors.

Microinvasion has not been consistently associated with an adverse prognostic effect [6, 34, 35]. A previous study evaluating various patterns of stromal-epithelial invasion not meeting the criteria for classic destructive invasion in a series of 60 SBT (FIGO I-III) found that neither size of the largest invasive aggregate (1–12 mm maximum dimension) nor extent of stromal involvement and number of microinvasive foci correlated with outcome [34]. In a meta-analysis [36], both micropapillary pattern and microinvasion were associated with higher recurrence rates (36%; 92/255 and 23%; 47/203, respectively) although it is not documented how many of these cases were associated with invasive peritoneal disease (LGSC, previously designated “invasive implants”). Other recent studies found no association of microinvasion with recurrence rate or survival [37, 38].

#### SBT—micropapillary variant

The micropapillary variant of SBT is now mentioned as a distinct subtype of SBT in the recent WHO classification, comprising 5–15% of SBT in different series [30, 39, 40]. This SBT variant was initially described as “non-invasive low-grade serous carcinoma” (non-invasive LGSC) by Kurman [39, 40], and this term has been adopted as synonymous with “SBT—micropapillary variant” in the current WHO classification [5]. The architectural criteria for its diagnosis have not changed. Micropapillary SBT is characterized by the lack of hierarchically branching papillae, showing either elongate filiform “micropapillae” (≥5:1 length to width ratio) or cribriform epithelium lining the cyst walls or large-caliber fibrovascular papillae, with at least one area of continuous micropapillary or cribriform growth >5 mm in one dimension [6, 25, 39, 40] (Fig. 1c to e). The stroma of micropapillary SBT is characterized by a higher fibroblast density compared to conventional SBT. In contrast to the previous WHO classification, additional cytologic criteria have been stated for the diagnosis of micropapillary SBT requiring “nuclear atypia greater than that allowed in SBT” typically characterized by rounded cells with lack of cilia, high nuclear to cytoplasmic ratio, and often small but prominent cherry-red nucleoli. SBT with a micropapillary pattern not meeting these diagnostic criteria (size >5 mm and increased cytologic atypia) should be classified “SBT with focal micropapillary features” [5].

The biologic nature of the micropapillary variant of SBT and its relation to conventional SBT and invasive LGSC, respectively, remains controversial. Kurman suggested the micropapillary variant of SBT as an intermediate entity in the progression from SBT to LGSC [17]. Gene expression analysis performed on laser-microdissected tumor cells from 37 cases of conventional SBT (*n* = 17), micropapillary variant of SBT (*n* = 9), and LGSC (*n* = 11) support this view, demonstrating differential gene expression patterns between SBT and its micropapillary variant, but no differences in gene expression between micropapillary SBT and LGSC [41]. All three entities are genomically relatively stable, and LGSC demonstrated only marginally increased chromosomal aberrations compared to SBT and the micropapillary variant of SBT [42–47].

A micropapillary pattern alone is no independent prognostic factor [6, 7], and only those cases associated with invasive peritoneal disease (LGSC, previously designated invasive implants) showed shorter disease-free and overall survival [37, 48, 49]. The largest nationwide cohort of SBT with central pathology review included 1487 women diagnosed with SBT or micropapillary variant of SBT in Denmark over a 25-year period (1978–2002) [50]. This study demonstrated that the overall survival of women with tumor confined to the ovaries (FIGO stage I) is not different from the general population. Overall survival was only reduced in women with advanced stages, and this applied to both women with SBT and the micropapillary variant of SBT [50]. However, the micropapillary variant of SBT more frequently presented at advanced stages compared to conventional SBT (27 versus 13%) and was more frequently associated with invasive peritoneal disease [50]. Conflicting data exist regarding the recurrence risk associated with the micropapillary variant of SBT. While some studies reported higher recurrence rates [51, 52], others including the largest multicenter study of BOT to date with 950 patients found no association with recurrence risk [7, 30, 37, 48].

The current clinical management of the micropapillary variant of SBT does not differ from conventional SBT. For histopathologic assessment of micropapillary SBT, thorough sampling is critical and particularly extra-ovarian lesions should be assessed for the possible presence of invasive disease (LGSC).

#### Low-grade serous carcinoma

The most important differential diagnosis of all SBTs is invasive LGSC. As mentioned in the previous section, the cytomorphology of LGSC is identical to the micropapillary variant of SBT (syn. non-invasive LGSC) and thus is not a discriminating criterion. Architecture and stroma, however, differ with a cell-rich, micropapillary or tubulus-like epithelial proliferation embedded in a collagenous, often hyalinized matrix with only scant fibroblasts and no significant inflammation.

#### Implants of SBT

Approximately one third of SBTs are associated with peritoneal implants [7, 30, 53]. The prior subdivision of non-invasive and invasive implants has been abandoned in the recent WHO classification, and any invasive foci are now considered peritoneal LGSC reflecting their similar biologic behavior [5].

Implants consist of serous epithelial proliferations on the peritoneal surface or in peritoneal invaginations, showing either (I) branching papillae covered by serous epithelial cells surrounded by a glomerulus-like small cyst and a calretinin-positive cell rim without a stromal response (previously called epithelial implant) or (II) tubular glands, small nests, and single eosinophilic cells within a desmoplastic inflamed “granulation tissue-type” stroma (previously called desmoplastic implant). Since both types of implants frequently occur together and their subclassification has no prognostic relevance, they are collectively designated “implants” in the WHO 2014 classification. By definition, implants are confined to the peritoneal surface without infiltration of the underlying subperitoneal fat. Of note, omental implants limited to the peritoneal surface can result in merging of lobular clefts, thereby imitating an infiltrative growth pattern [6, 25, 40].

In contrast, peritoneal lesions characterized by an extensive epithelial component with haphazardly arranged glands, solid nests, and/or papillary structures accompanied by desmoplastic stroma with little or no inflammation and with invasion of underlying subperitoneal tissue or omental fat were classified invasive implants in the previous WHO classification. The current WHO 2014 classification now designates these foci as LGSC. In addition, implants lacking an infiltrative growth but displaying other features suggestive of LGSC, particularly a micropapillary or cribriform growth pattern and clear retraction spaces, should also be designated LGSC.

This new nomenclature of extra-ovarian invasive disease is supported by studies demonstrating their similar biologic behavior and disease progression compared to LGSC [7, 54]. Nevertheless, the volume of invasive disease may have prognostic impact. Future studies are needed to clarify the long-term outcome of ovarian SBT associated with small foci of invasive peritoneal disease (LGSC) compared to primary ovarian/peritoneal LGSC presenting with widespread peritoneal carcinomatosis and bulky disease. The size of invasive foci should be stated in the pathology report*.*

Most studies reported no adverse prognosis for (non-invasive) implants, whereas invasive peritoneal disease (LGSC) was associated with shorter overall survival [12, 25, 55] [7]. In a meta-analysis of 97 studies including 4129 patients with SBT, Seidman et al. reported an overall survival of virtually 100% for stage I tumors and 95.3% for advanced stage tumors with (non-invasive) implants, whereas survival for tumors associated with invasive peritoneal disease (LGSC) was reduced to 66%. The presence of a micropapillary variant of SBT was a strong predictor for concurrent invasive peritoneal disease (LGSC) [7].

Lymph nodes may also contain foci of SBT similar to their peritoneal counterparts, with individual or clusters of serous epithelial cells with intense eosinophilic cytoplasm located within sinuses, most commonly in subcapsular location. These intrasinusoidal cells have recently been shown to display a senescent phenotype, similar to the epithelial cells of microinvasive foci, and have no prognostic effect [33]. Rarely, foci of LGSC with associated desmoplasia and destruction of lymph node architecture have been reported in patients with ovarian SBT and should be classified as LGSC [5].

According to both UICC and FIGO classification, the T-stage is affected by the presence of peritoneal implants, similar to the staging of invasive carcinoma [56]. In contrast, lymph node involvement by SBT is not considered metastatic disease and classified as pN0, and the benign nature of these lesions should be mentioned in the pathology report [5].

### Mucinous borderline tumor

**Fig. 2:**
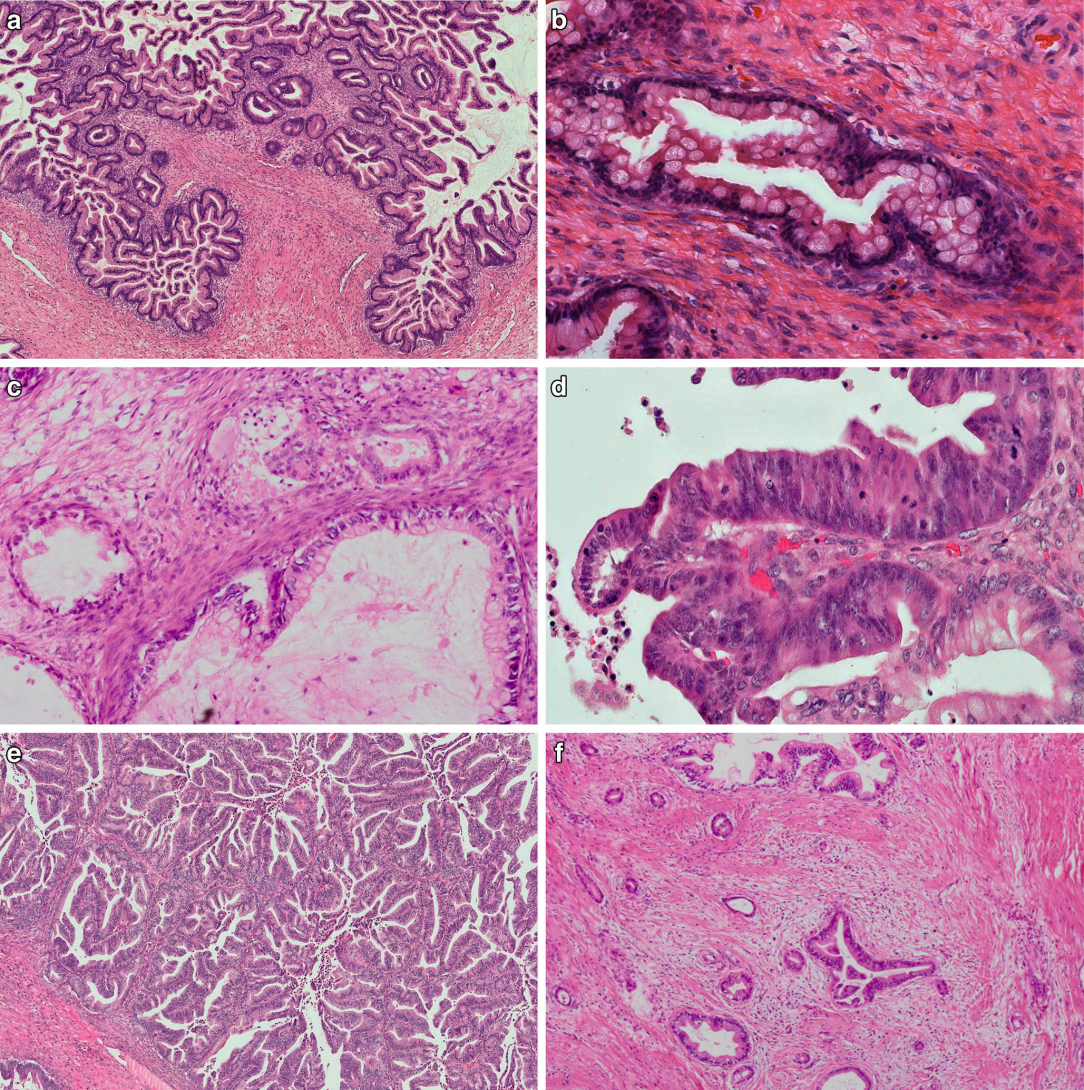
MBT showing cystic glandular structures with papillary infoldings, columnar cells with abundant cytoplasmic mucin, admixed with goblet cells of variable degrees of maturation (**a**) and with basally located nuclei with no considerable nuclear atypia (**b**). MBT with microinvasion is characterized by small cell groups and glands with cytoplasmic eosinophilia within a normal ovarian stroma without desmoplastic change (**c**). MBT with intraepithelial carcinoma is characterized by focal high-grade nuclear atypia, commonly associated with more complex epithelial proliferations, next to conventional MBT structures with sharp transition (**d**). Mucinous carcinoma with expansile (“pushing border”) invasion shows confluent glandular and papillary epithelial proliferations without stromal desmoplasia (**e**). Mucinous carcinoma with destructive invasion demonstrates haphazardly infiltrating glands and is characterized by desmoplastic tumor stroma (**f**)

Mucinous borderline tumors (synonymous atypical proliferative mucinous tumors) are the second most common type and account for about 35 to 45% of ovarian borderline tumors [5]. These tumors are usually large, unilateral, and cystic with a smooth ovarian surface, composed of multiple cystic spaces with variable diameter. The cysts are lined by columnar mucinous epithelium of gastric or intestinal differentiation, with papillary or pseudopapillary infoldings, and admixed goblet cells and neuroendocrine cells [57] (Fig. 2a). The nuclei are basally located, isomorphic, and with evenly distributed chromatin [58, 59] (Fig. 2b). Mucinous cystadenomas are characterized by a similar mucinous epithelium but lack papillary infoldings. At least 10% of the epithelial volume must demonstrate increased proliferation with papillary infoldings or pseudostratification and mild to moderate nuclear atypia to qualify as mucinous borderline tumor (MBT)). Immunohistochemically, MBTs are characterized by their non-Mullerian differentiation with absence of WT1, estrogen and progesterone receptor expression [60, 61]. Most tumors demonstrate diffuse expression of cytokeratin 7 with patchy co-expression of cytokeratin 20 and variable (usually weak) expression of CDX2 in approximately 40% of cases [57, 62, 63]. While limited previous studies had only assessed PAX8 expression in mucinous carcinomas (10/25; 40% expression) [64], a recent study confirmed expression of PAX8 in MBT (14/23; 61%) as well as ovarian mucinous carcinomas (11/24; 46%). HER2 overexpression or amplification has been reported in up to 20% of MBT [65] and may sometimes be useful in distinguishing primary from metastatic ovarian involvement. The proliferative rate is usually low, and Ki67 demonstrates predominant expression in cells at the base of the papillary structures with decreasing expression toward their tips.

No unequivocal cases of peritoneal implants associated with MBT have been reported in the literature, and their occurrence should prompt exclusion of secondary ovarian involvement (see below discussion on metastatic disease). The vast majority of MBTs have an excellent prognosis with overall survival approaching 95–100% [5]

**Fig. 3:**
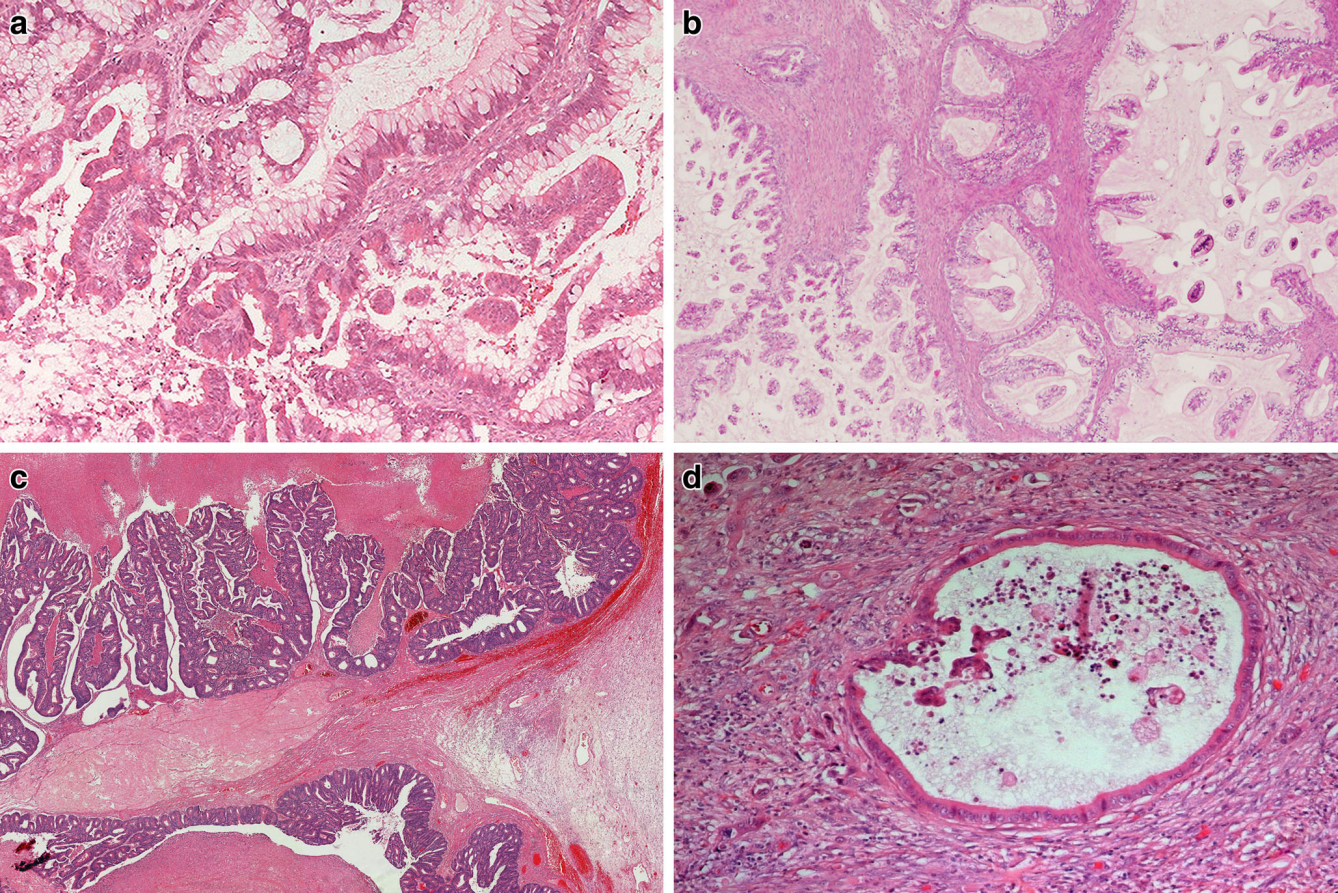
Ovarian metastases mimicking MBT originating from various extra-ovarian primary tumors: metastatic low-grade appendiceal mucinous neoplasm (**a**), gastric intestinal type (**b**), colorectal (**c**), and pancreatic ductal adenocarcinoma (**d**)

. A recent single-center study including 254 patients with stage I BOT found a higher incidence of invasive recurrences in MBT compared to SBT following fertility-conserving surgery. However, possible insufficient sampling is a concern in large MBT, and this finding needs to be confirmed in further studies [66]. Histogenetically, development of MBT from mature teratoma or via metaplastic Brenner tumors has been discussed, and ovarian mucinous and Brenner tumors may share differentiation from a common stem cell [67, 68, 69, 70]. The most common molecular genetic aberrations are KRAS mutations previously observed in approximately 60% of MBT, with detection frequencies of up to 92% by newer targeted deep sequencing approaches [71–73]. Ovarian mucinous tumors are markedly heterogeneous, with frequent co-occurrence of adenomatous, borderline, and carcinomatous components, suggesting a stepwise progression in at least part of the cases. Therefore, careful gross examination and sampling is mandatory and at least one section per centimeter largest tumor diameter should be examined, increasing to two blocks per centimeter diameter in mucinous tumors >10 cm [5]

**Fig. 4:**
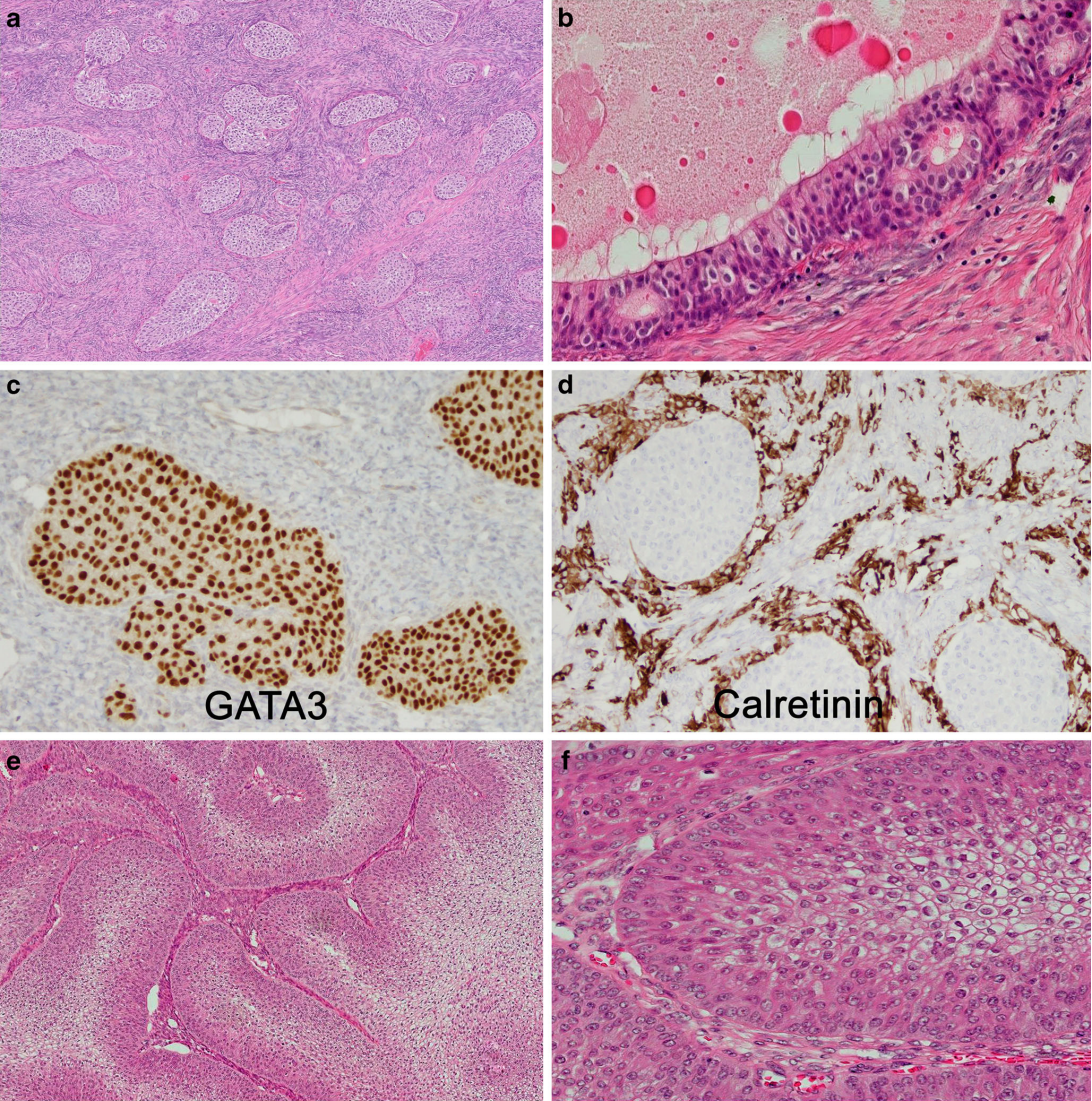
Brenner tumor (**a**) showing epithelial cell nests of variable size, with transitional cell-like morphology, embedded in a fibrous stroma. The epithelium-to-stroma ratio is even. Central cysts are lined by a single layer of columnar mucinous cells. Metaplastic Brenner tumor (**b**) demonstrates a cystic structure with predominance of mucinous epithelium. GATA3 (**c**) is diffusely expressed in Brenner tumors, and sometimes many luteinized stromal cells are present highlighted by calretinin stain (**d**). Brenner BOT are characterized by a significantly increased epithelium-to-stroma ratio (**e**) but share the same cytological details as benign Brenner tumor (**f**)

.

#### Microinvasion

MBT with microinvasion is defined by stromal invasion measuring less than 5 mm in the greatest linear dimension and consisting of single cells, clusters, or small foci of confluent glandular or cribriform growth, regardless of the number of microinvasive foci (Fig. 2c). Cases with microinvasive foci displaying high-grade nuclear atypia should be designated microinvasive carcinoma according to the recent WHO classification, although the prognostic value of this category remains to be defined [5, 38]. Microinvasion has been reported in 4 to 18% of MBT and has no adverse effect on prognosis [74–76]. Nevertheless, additional sampling as well as immunohistochemical testing are recommended to exclude frankly invasive carcinoma or metastatic disease.

#### Intraepithelial carcinoma

MBT with intraepithelial carcinoma has been described in 40 to 55% of MBT and is characterized by areas with high-grade nuclear atypia that differ cytologically from the background epithelium, usually with sharp demarcation [74–76] (Fig. 2d). While intraepithelial carcinoma is often associated with increased architectural complexity of epithelial stratification or cribriform growth, this criterion is neither necessary nor sufficient, and the diagnosis of intraepithelial carcinoma should be solely based on nuclear cytomorphology. Although some studies reported a higher recurrence risk, most studies observed no difference in overall survival in cases of MBT with or without intraepithelial carcinoma [75, 77, 78].

#### Mural nodules in MBT

A peculiarity of MBT is their occasional association with so-called mural nodules, comprising either reactive sarcoma-like nodules or foci of frank sarcoma or anaplastic carcinoma within the cyst wall, varying widely in size (up to 10 cm) and number. Sarcoma-like mural nodules have no adverse prognostic impact [5]. A study including 34 patients with mucinous ovarian tumors and nodules of anaplastic carcinoma found that the presence of anaplastic nodules in unruptured stage I mucinous tumors did not carry an adverse prognosis [79]. MBT with anaplastic carcinoma should be classified according to its carcinomatous component.

#### Differential diagnosis of MBT and mucinous carcinoma with expansile or destructive invasion

The distinction of MBT from its invasive counterparts, in particular those with a confluent/expansile growth pattern, can be challenging and is worth emphasizing here. The existence of a subgroup of mucinous ovarian neoplasms lacking the criteria for classic destructive stromal invasion but rarely associated with adverse prognosis and metastasis has long been recognized. In 1973, Hart and Norris first suggested a category of “non-invasive mucinous carcinoma” of the ovary defined by stratification of more than three cell layers and severe nuclear atypia [80]. The current WHO classification distinguishes two types of mucinous carcinoma based on their growth pattern [5]:

*Mucinous carcinoma with confluent/expansile invasion* is characterized by a confluent growth pattern, marked epithelial proliferation with glandular crowding, and solid or cribriform epithelial sheets with “labyrinthine appearance” obliterating the cystic spaces. The degree of nuclear atypia is often similar to MBT, and there is usually a sharp tumor-host interface without stromal desmoplasia [76]. Prognosis of this tumor variant, especially when confined to the ovary at presentation, is very favorable and the overall survival appears to approach that of MBT [75, 78, 81].

*Mucinous carcinoma with destructive infiltrative invasion* is less common and defined by obvious invasive growth with a haphazard arrangement of infiltrative glands, tubules, or epithelial cell nests [76]. Associated stromal desmoplasia is commonly present, but its absence does not exclude the diagnosis of infiltrative carcinoma (Fig. 2f). The majority of mucinous carcinomas are diagnosed at an early stage when confined to one ovary (FIGO stage I). Prognosis for stage I disease is very favorable, though not as good as for expansile mucinous carcinomas. Primary advanced stage mucinous carcinomas are rare, associated with very poor prognosis, and should prompt exclusion of metastatic disease from an extra-ovarian primary [82, 83].

#### Differential diagnosis of MBT and secondary ovarian involvement by metastatic disease

No single criterion allows definitive differentiation of primary versus metastatic ovarian mucinous tumors, but by taking into account combined clinical, histological, and immunohistochemical features, distinction is possible in more than 85% of cases.

Features favoring metastases include smaller size <10 cm, bilaterality, surface involvement, (multi)nodular growth pattern, extra-ovarian disease, and associated pseudomyxoma ovarii or pseudomyxoma peritonei. Cytomorphologic features raising suspicion for metastasis in cases with typical MBT architecture include foci of high-grade nuclear atypia, prominent nucleoli, and significant mitotic activity. In contrast, a primary ovarian MBT is supported by associated mucinous cystadenofibroma, Brenner tumor, teratoma, or endometriosis [84–88].

Ovarian metastases with mucinous differentiation arise most frequently from appendiceal primary tumors, in particular low-grade appendiceal mucinous neoplasms (LAMN) [86] (Fig. 3a). Consequently, most treatment guidelines recommend routine appendectomy in cases of MBT or mucinous carcinoma even if the vermiform appendix appears unremarkable intraoperatively. Metastases from appendiceal primaries usually show diffuse expression of cytokeratin 20, CDX2, and MUC2; variable expression of MUC5AC; and patchy co-expression of cytokeratin 7 in approximately half of the cases. Most low-grade appendiceal mucinous neoplasms are associated with the clinical picture of pseudomyxoma peritonei [57, 67]. In contrast, only a small percentage of those MBTs arising within mature cystic teratoma have been unequivocally associated with pseudomyxoma peritonei [67, 85]. Of note, teratoma-associated MBT are characterized by an immunohistochemical expression profile similar to mucinous neoplasms of the lower gastrointestinal tract, with diffuse expression of cytokeratin 20 and CDX2 and absence of cytokeratin 7 [63, 89, 90].

Intense and diffuse expression of CDX2 should raise suspicion for metastasis from a gastrointestinal primary, with the rare exception of teratoma-associated MBT [63, 89].

The second most common mimics of MBT are metastatic mucinous carcinomas of pancreatobiliary origin [86] (Fig. 3d), often demonstrating a morphological pattern of small invasive single cells or glands with marked atypia next to large cytologically bland cystic structures. Their immunohistochemical expression profile of cytokeratins 7 and 20 and MUC5AC is comparable to MBT, with additional expression of cytokeratin 17 and MUC1, as well as expression of cadherin-17 and loss of DPC4 in about 50% of cases [57, 91, 92]. Negativity of DPC4 excludes an ovarian primary while expression of PAX-8 strongly favors an ovarian primary. Of note, a small subset of non-mucinous pancreatic adenocarcinomas (1/12; 8%) as well as two out of two cholangiocarcinomas demonstrated PAX8 expression in a comprehensive analysis [64, 93].

Further extra-ovarian primaries giving rise to mucinous ovarian metastases include colorectal (Fig. 3c), gastric (Fig. 3b), breast, and endocervical carcinomas. While the expression pattern of cytokeratins 7 and 20 is most useful in the differential of MBT and lower gastrointestinal tract tumors, with diffuse expression of cytokeratin 20 and absence of cytokeratin 7 favoring gastrointestinal origin, this panel is not helpful for distinguishing MBT from upper gastrointestinal tract neoplasms. In contrast, absence of CDX2 strongly favors primary ovarian origin over both upper and lower gastrointestinal tract origins [63, 94]. Additional immunohistochemical markers that have been suggested in this context include the expression of cadherin-17, racemase, and nuclear ß-catenin, in the absence of cytokeratin 7 in tumors of gastrointestinal origin [57, 92, 95]. Metastases from gastric carcinomas can be difficult to prove by immunohistochemistry; approximately half of the cases demonstrate expression of cadherin-17, and a quarter express racemase, which is absent in almost all primary ovarian mucinous tumors [92, 96]. Further potentially useful immunohistochemical markers include estrogen and progesterone receptors and GATA3 for distinction of metastases from breast carcinomas and p16 and HPV for metastatic cervical adenocarcinomas.

### Borderline Brenner tumor

Transitional cell/Brenner tumors of the ovary are generally rare, and only less than 3–5% are of borderline or invasive type. To date, approximately 30 borderline Brenner tumors have been reported in the literature [5, 97].

Borderline Brenner tumors are thought to arise from benign Brenner tumors, as both components often occur together. Commonly found Walthard cell nests have been suggested as possible histogenetic origin based on their shared immunohistochemical expression profile, but are only infrequently associated with Brenner tumors [68, 98]. Limited data on their molecular characteristics suggested that loss of CDK2A (gene encoding p16) and somatic mutations in KRAS and PIK3CA may be involved in the progression from benign to borderline Brenner tumors [99, 100]. Data on their biologic behavior is limited due to the small number of reported cases. Despite a generally favorable prognosis, rare recurrences and deaths have been reported, including one uterine recurrence of a borderline Brenner tumor harboring an exon 9 PIK3CA mutation [97].

Compared to benign Brenner tumors which are commonly small (<2 cm) and predominantly solid/fibromatous (Fig. 4a), borderline Brenner tumors are usually larger than 10 cm (mean 18 cm) with a predominating epithelial proliferation (Fig. 4e and f). Cystic areas demonstrate papillary or polypoid infoldings, covered by a thick layer of transitional-type cells, resembling non-invasive papillary urothelial carcinomas of the urinary tract. Mitotic figures may be numerous.

The immunohistochemical expression profile of Brenner tumors, including benign, borderline, and malignant, overlaps with urothelial differentiation, with expression of GATA3 (Fig. 4c), cytokeratin 7, and p63; variable expression of uroplakin and thrombomodulin; and absence of WT1 and estrogen and progesterone receptors. Calretinin may highlight luteinized stromal cells. (Fig. 4d) In contrast to urothelial differentiation, cytokeratin 20 is usually absent [98, 101].

An important differential diagnosis of borderline Brenner tumor is (benign) metaplastic Brenner tumors, where the solid transitional cell nests are replaced by mucinous differentiation (often expressing cytokeratin 20) with a central cystic cavity (Fig. 4b) and sometimes associated with complex glandular proliferations [102]. As mentioned above, it is possible that such biphasic mucinous-transitional cell neoplasms may represent divergent differentiation from a common stem cell. Malignant Brenner tumors of the ovary are by definition associated with a benign or borderline Brenner tumor and distinguished by an invasive component usually resembling high-grade invasive urothelial carcinoma. In the differential diagnosis with metastatic urothelial carcinoma of the urinary tract, the detection of an associated benign Brenner tumor component, as well as expression of CA125 and absence of cytokeratin 20, may be helpful to confirm a borderline Brenner tumor of primary ovarian origin.

### Borderline ovarian tumors related to ovarian endometriosis

Endometriosis affects 6–10% of reproductive-age women and has been associated with specific molecular and developmental abnormalities that allow endometrial cells to grow outside of the uterus following menstrual efflux, epithelial metaplasia, or differentiation of stem cells [103, 104]. Endometriosis can be separated into superficial peritoneal and deep infiltrating subgroups. Ovarian endometriosis and in particular ovarian endometriotic cysts represent examples of the second group and have been shown to be clonal lesions by many but not all investigators [105]. Endometriotic cysts have also been associated with increased levels of oxidative stress and reactive oxygen detoxification pathway intermediates, specific cytogenetic abnormalities, loss of PTEN and ARID1A expression, microsatellite instability, let-7 microRNA-dysregulated overexpression of KRAS, and significant epithelial atypia (seen in 8% of cases) [105–109]. The last feature, known as atypical endometriosis, is frequently seen adjacent to endometriosis-associated carcinomas and BOT as detailed below [110].

### Seromucinous borderline tumor

Seromucinous borderline tumor (SMBT), also known as atypical proliferative seromucinous tumor, formerly endocervical-type mucinous BOT, or mullerian mucinous BOT, accounts for approximately 5–7% of all BOT. Although long recognized under a variety of names, these tumors are now formally established as a separate category in the revised 2014 WHO Classification of Tumours of the Female Genital Organs. Of note, the term mullerian mucinous BOT or mixed mullerian BOT is more recently being favored by some pathologists as it reflects the mullerian differentiation of these tumors [111].

**Fig. 5:**
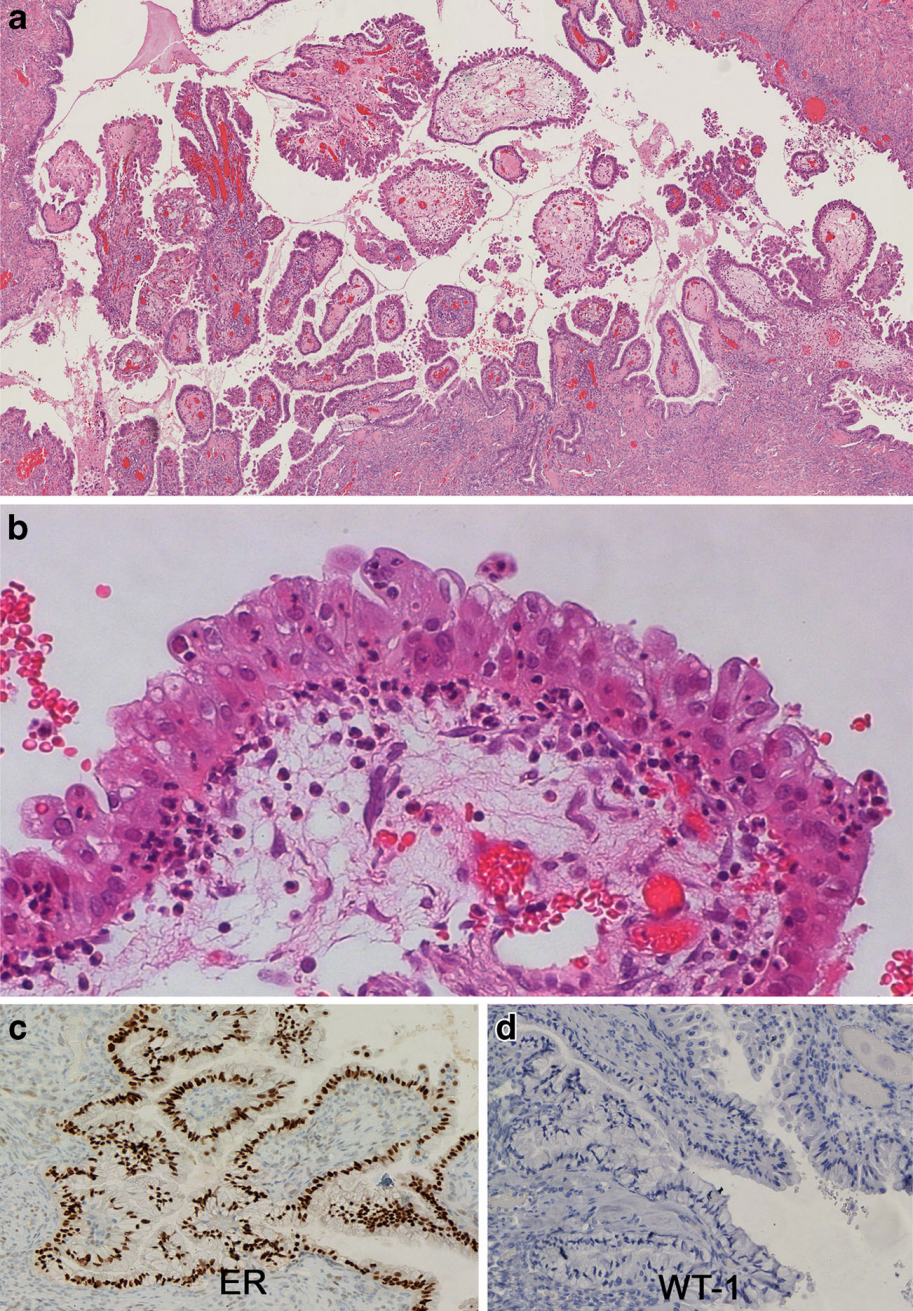
SMBT demonstrating a papillary architecture with hierarchical branching (**a**). The epithelium is columnar with focal multilayering with papillary and pseudopapillary infoldings and variable cytoplasmic mucin content. Stroma and epithelium show infiltration by neutrophils (**b**). Immunohistochemical expression of ER (and/or PR) (**c**) and absence of WT1 (**d**) is typical

Unlike the other endometriosis-associated BOT (discussed below), SMBT is the most common type of tumor within its category far exceeding benign seromucinous tumors (cystadenoma and adenofibroma) and seromucinous carcinomas [112]. SMBTs usually arise in young women (34–44 years old) and present as unilocular or paucilocular cysts averaging 8–10 cm in diameter often with intracystic papillae [113, 114]. Bilateral involvement is seen in 40%, and up to 20% have peritoneal implants or lymph node involvement. Associated endometriosis is seen in 30–70% of cases, and SMBT often co-exists with endometriosis-related cancers, most commonly of endometrioid histology, so extensive sectioning is indicated. It has been proposed that SMBTs usually arise within atypical endometriotic cysts that undergo mucinous differentiation. As in other endometriosis-related BOT, loss of ARID1A expression is common and mutations have been documented in 33% of cases [115, 116]. Similar to other mucinous female genital tract tumors, KRAS mutations are frequent, being detected in 69% of cases [117]. Histologically, SMBTs have architectural features similar to SBTs (Fig. 5a). However, the branching papillae in SMBTs are lined by varying proportions of endocervical-type mucinous, tubal-type serous, endometrioid, and indeterminate cells with dense eosinophilic cytoplasm. Hobnail or clear cells and prominent squamous metaplasia may also be seen. The presence of at least two different types of mullerian differentiation is required for the diagnosis [5]. Virtually all cases are associated with a significant amount of acute inflammation, edema, and occasional eosinophils which can be diagnostically useful (Fig. 5b). Unlike most other BOT, the majority of cases do stain positively for vimentin. Estrogen and progesterone receptors are generally positive (Fig. 5c), and WT1 (Fig. 5d), CK20, and CDX2 are negative. Diffuse cytoplasmic expression of MUC5AC is seen in the endocervical-type mucinous component [59, 60, 118]. Microinvasion and intraepithelial carcinoma may be seen and are defined in the same way as in other BOT (see above). Peritoneal implants associated with SMBT have been infrequently reported. Most cases have a good outcome, even in the presence of extraovarian disease. Destructive stromal invasion of 5 mm or more, complex expansile growth, and/or invasive extraovarian disease are the standard criteria defining progression to seromucinous carcinoma [112].

### Endometrioid borderline tumor

**Fig. 6:**
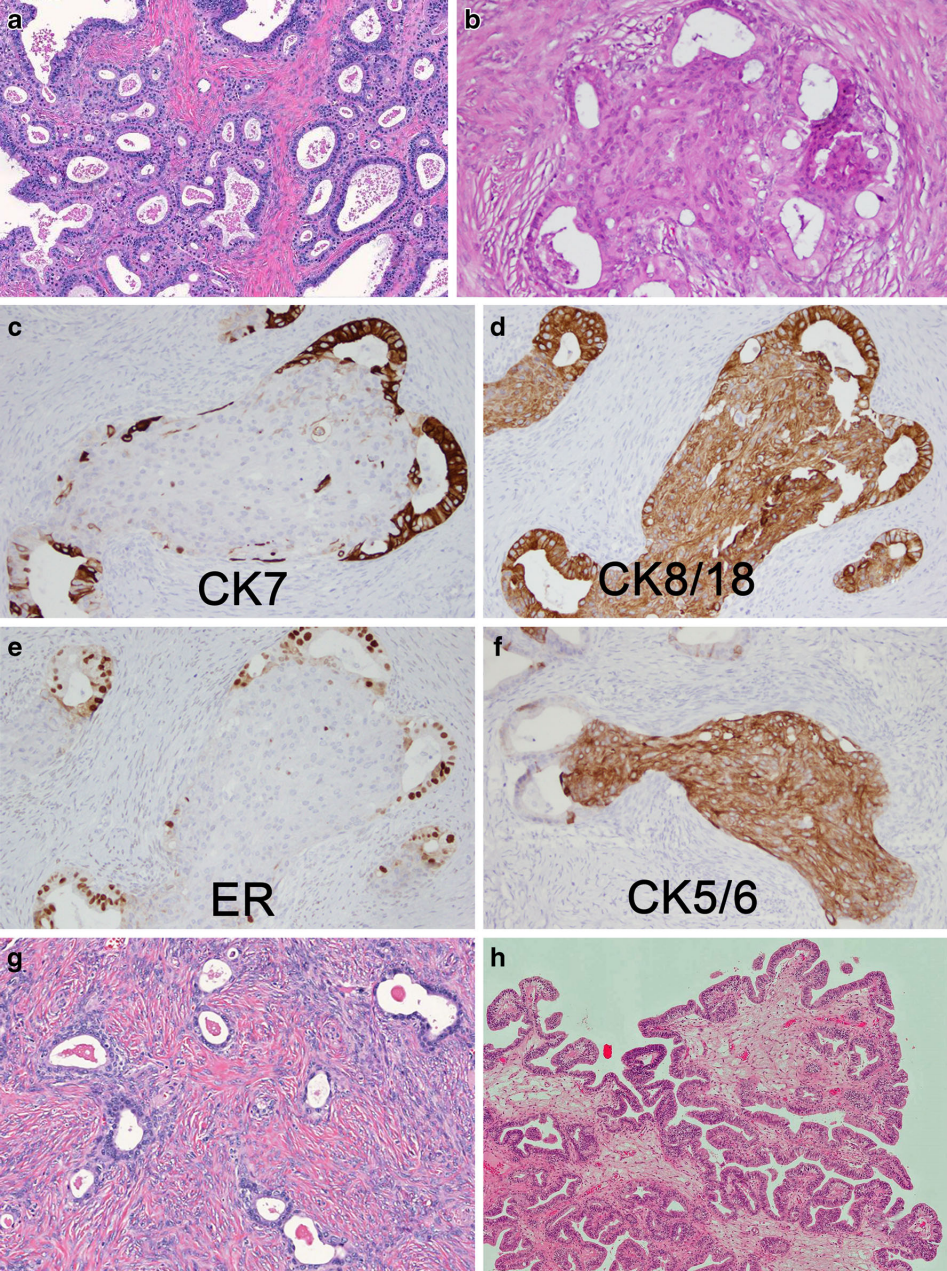
EBT of adenofibromatous type with endometrioid glandular proliferations embedded in a fibroblastic stroma (**a**) with some epithelial cell nests consisting of a central squamous area (**b**). Glandular proliferations express CK7 (**c**) and ER (**e**). CK 8/18 is diffusely expressed (**d**) whereas the squamous areas are CK5/6-positive (**f**) and do also express CDX2 (not shown). Endometrioid cystadenofibroma (**g**) is characterized by round and cystic glands, evenly distributed in a fibroblastic stroma. Cystic/villoglandular EBTs (**h**) share the same architecture as SBTs but differ in their endometrioid cytology

Endometrioid borderline tumor (EBT), also known as atypical proliferative endometrioid tumor, accounts for 2–3% of BOT [119, 120]. Mean age at diagnosis is 57 years. Co-existing endometriosis is seen in 63% of cases, and 39% have synchronous hyperplasia or carcinoma in the endometrium. Therefore, endometrial curettage is recommended in case of fertility preserving therapy. ARID1A mutations are detected in 40% of cases [115]. KRAS mutations are found in 29% of cases with associated endometriosis, but are rare in the remainder [121]. Abnormalities in the Wnt/beta-catenin and PI3K/mTOR pathways are also common. Average tumor diameter is 9 cm with two thirds of cases having a cystic component and the remainder being predominantly solid. An adjacent benign endometrioid adenofibroma is observed in 50% of cases. Bilaterality is seen in 4% of cases. Histologically, tumors can show two patterns, adenofibromatous or villoglandular. Both subtypes may show focal areas of cribiform growth, squamous morules, and intermixed necrotic debris. In the adenofibromatous subgroup, distinction from adenofibroma is made on the basis of a complex hyperplastic epithelial growth pattern with or without mild to moderate nuclear atypia (Fig. 6a and b). Villoglandular EBT may occasionally demonstrate an architecture similar to SBT but differ in their cytologic features (Fig. 6h). The cells are more cylindrical with oval nuclei orientated perpendicular to the basement membrane. Sometimes, this can be ambiguous and there may be overlap with serous differentiation. Immunohistochemical stains (Fig. 6c to f) are not usually necessary to differentiate EBT from SBT. However, WT1 is usually negative in contrast to SBT, while focal p16 staining may be seen in up to 50% of EBT [122]. Intraepithelial carcinoma (severe nuclear atypia) and microinvasion follow the same criteria as in MBT, but have little or no prognostic significance. Peritoneal implants are exceedingly rare [119]. Progression to carcinoma is defined by a confluent expansile growth and/or one or more foci of destructive invasion of ≥5 mm. In view of its rarity, absence of extraovarian disease, and the lack of any well-documented malignant behavior, the diagnosis of EBT currently has few if any implications for clinical management [123]

.

Mimics of EBT include metastases from gastrointestinal, endocervical, or endometrial adenocarcinomas. Positivity of PAX8 and estrogen receptor distinguish EBT from gastrointestinal carcinoma. Diffuse and strong nuclear p16 expression and detection of HPV-DNA may be helpful for the differentiation between EBT and metastasis of HPV-related endocervical adenocarcinoma. MUC4 and HIK1083 can be useful for distinguishing HPV-negative endocervical adenocarcinomas of gastric type. Metastases from well-differentiated endometrial carcinomas demonstrate a similar immunohistochemical expression pattern as EBT.

### Clear cell borderline tumor

**Fig. 7:**
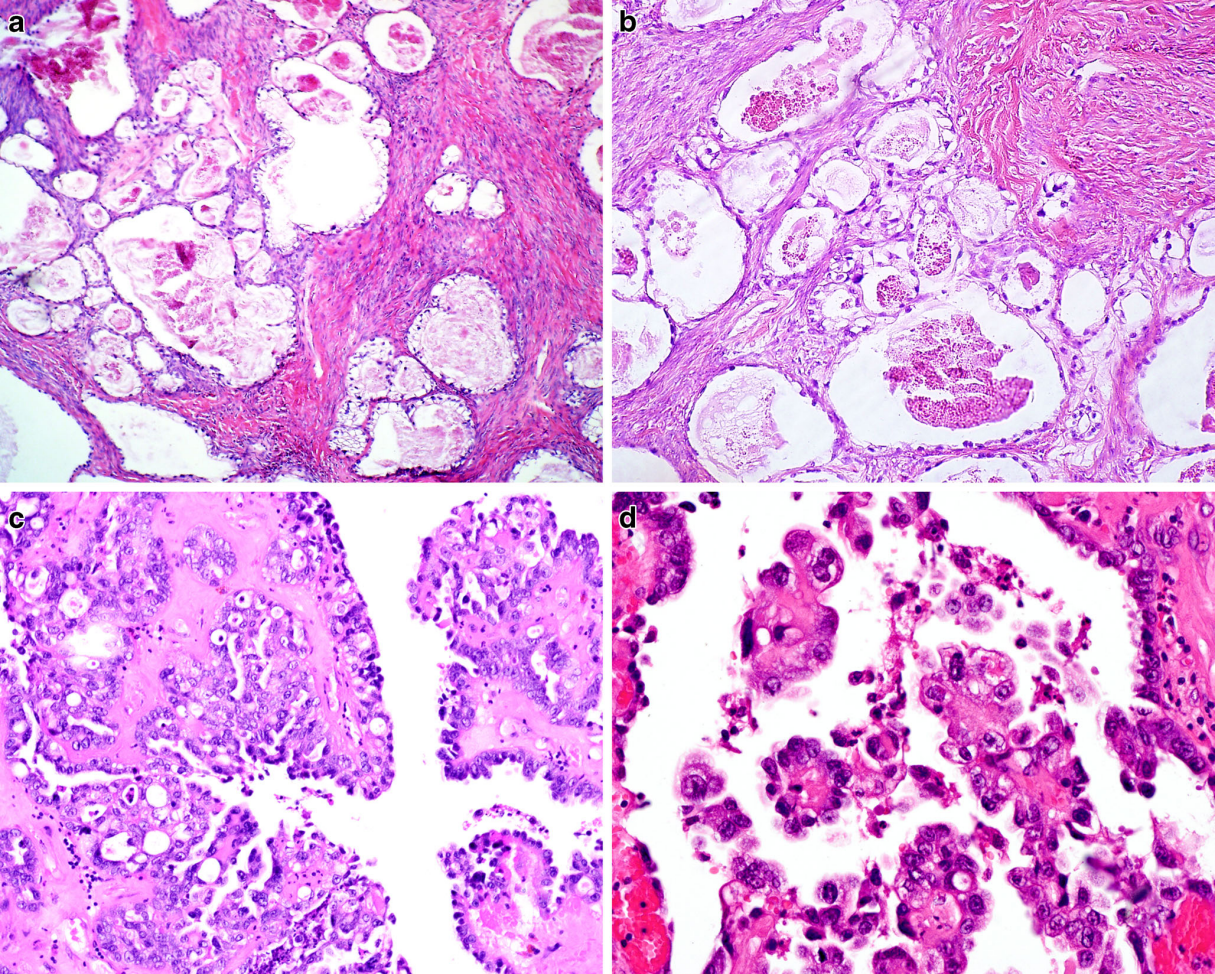
CCBT demonstrating variably sized glands lined by clear cells, evenly spaced with focal glandular crowding, and embedded in a fibromatous stroma (**a**), with mild to moderate nuclear atypia (**b**). In contrast, intracystic clear cell carcinoma demonstrates intracystic papillary proliferations lined by clear and hobnail cells (**c**) with high-grade nuclear atypia and prominent nucleoli (**d**)

Clear cell borderline tumor (CCBT), also known as atypical proliferative clear cell tumor, represents less than 1% of all BOT [5, 124, 125]. It usually occurs between 59 and 68 years of age. Tumors average 6 cm in diameter and are generally unilateral. The majority have a solid appearance with small cysts (“swiss cheese” pattern), similar to clear cell adenofibroma but with some softer, fleshier areas. A minority arise within atypical endometriotic cysts. CCBTs are highly likely to be associated with foci of frank clear cell carcinoma, so extensive sectioning, preferably submitting the entire specimen, is recommended. Given its rarity, few specific molecular studies have been conducted, but there is a strong association with endometriosis, often atypical [110]. When seen adjacent to clear cell carcinoma, a shared loss of ARID1A expression suggests a clonal relationship [126]. The frequency of PIK3CA mutations, an early event in clear cell carcinoma, has not been studied in adenofibromatous-type CCBT. Histologically, CCBTs are characterized by round to oval evenly spaced glands embedded in an adenofibromatous-type stroma (Fig. 7a). Compared with clear cell adenofibroma, there is more glandular crowding and proliferation. Glands are lined by flat, cuboidal, or hobnail cells with moderate nuclear atypia (Fig. 7b), small nucleoli, and coarse chromatin clumping. Mitotic rate should be less than four per high power field. The key discriminating feature is the degree of nuclear atypia. Low-grade atypia characterizes adenomas whereas an intermediate nuclear grade defines the borderline category. Clear cell carcinoma is characterized by at least focal high-grade nuclear atypia with prominent nucleoli (Fig. 7d), commonly in a background of intermediate nuclear atypia, and often associated with hyalinized stroma [127]. However, nuclear grading is highly subjective and diagnosis should err on the side of malignancy, particularly in the tubulocystic variant which can often appear deceptively bland. Similarly, a purely cystic growth pattern is very unusual in CCBT and should raise suspicion for the intracystic variant of clear cell carcinoma (Fig. 7c). Immunostains for HNF1 and Napsin A (usually positive) and WT1 and estrogen and progesterone receptors (usually negative) may be helpful to establish clear cell differentiation in some cases. Microinvasion is defined similarly to other BOT (<5 mm). Although it has been stated that CCBT may show intraepithelial carcinoma, this diagnosis should be made with great discretion in view of the frequency of co-existent clear cell carcinoma. All CCBTs followed thus far have acted in a benign fashion. However, there is no data regarding the prognosis of cases with either intraepithelial carcinoma or microinvasion.

## Conclusion and perspective

In line with our evolving understanding that different ovarian cancer histotypes represent distinct disease entities, emerging knowledge suggests that subtypes of borderline ovarian tumors likewise comprise distinct biologic, pathogenetic, and molecular entities [17, 128]

precluding a single unifying concept for BOT. The borderline malignant potential is best validated for SBT which share molecular and genetic alterations with low-grade serous carcinomas and can present at higher stages with peritoneal implants and/or lymph node involvement. In addition, recurrences occur in a small percentage of SBT with occasional malignant transformation. On the other hand, non-serous subtypes of BOT commonly present at stage I confined to the ovary(ies) and are associated with an overall survival approaching that of the general population in well-sampled tumors.

Although a workshop sponsored by the NIH in 2003 provided consensus for many of the currently accepted diagnostic criteria of BOT [6], some challenges remain, e.g., distinction of MBT from expansile mucinous carcinoma or differentiation of SBT with microinvasion versus small foci of low-grade serous carcinoma. One of the most important changes of the current WHO 2014 classification is the new terminology of non-invasive implants associated with SBT, whereas any invasive foci (prior invasive implants) are now considered peritoneal LGSC more in line with their biological behavior.

Ongoing controversies include the terminology of non-serous borderline tumors as some pathologists prefer the term “atypical proliferative tumor” in view of their largely benign behavior. The concepts of intraepithelial carcinoma and microinvasion may evolve in the future as their presence appears to have no prognostic impact and is subject to considerable inter-observer variability.

Future studies such as large multicenter trials with associated molecular analyses should address these controversies and aim to identify reliable risk factors for recurrence or malignant transformation of SBT.
